# Double bonds of unsaturated fatty acids differentially regulate mitochondrial cardiolipin remodeling

**DOI:** 10.1186/s12944-019-0990-y

**Published:** 2019-02-14

**Authors:** Hsiu-Chi Ting, Li-Tzu Chen, Jo-Yu Chen, Yi-Li Huang, Rui-Cheng Xin, Jui-Fen Chan, Yuan-Hao Howard Hsu

**Affiliations:** 1 Department of Chemistry, Tunghai University, No.1727, Sec4, Taiwan Boulevard, Xitun District, Taichung, 40704 Taiwan, Republic of China; 2 Life Science Research Center, Tunghai University, No.1727, Sec4, Taiwan Boulevard, Xitun District, Taichung, 40704 Taiwan, Republic of China

**Keywords:** Cardiolipin, 18-carbon unsaturated fatty acids, Mitochondrial membrane composition, Mass spectrometry

## Abstract

**Background:**

Supplemented fatty acids can incorporate into cardiolipin (CL) and affect its remodeling. The change in CL species may alter the mitochondrial membrane composition, potentially disturbing the mitochondrial structure and function during inflammation.

**Method:**

To investigate the effect of the unsaturation of fatty acids on CL, we supplemented macrophage-like RAW264.7 cells with 18-carbon unsaturated fatty acids including oleic acid (OA, 18:1), linoleic acid (LA, 18:2), α-linolenic acid (ALA, 18:3), γ-linolenic acid (GLA, 18:3), and stearidonic acid (SDA, 18:4). Mitochondrial changes in CL were measured through mass spectrometry.

**Result:**

Our data indicated that OA(18:1) was the most efficient fatty acid that incorporated into CL, forming symmetrical CL without fatty acid elongation and desaturation. In addition, LA(18:2) and ALA(18:3) were further elongated before incorporation, significantly increasing the number of double bonds and the chain length of CL. GLA and SDA were not optimal substrates for remodeling enzymes. The findings of RT-qPCR experiments revealed that none of these changes in CL occurred through the regulation of CL remodeling- or synthesis-related genes. The fatty acid desaturase and transportation genes—*Fads2* and *Cpt1a*, respectively—were differentially regulated by the supplementation of five unsaturated 18-carbon fatty acids.

**Conclusions:**

The process of fatty acid incorporation to CL was regulated by the fatty acid desaturation and transportation into mitochondria in macrophage. The double bonds of fatty acids significantly affect the incorporation process and preference. Intact OA(18:1) was incorporated to CL; LA(18:2) and ALA(18:3) were desaturated and elongated to long chain fatty acid before the incorporation; GLA(18:3) and SDA(18:4) were unfavorable for the CL incorporation.

## Background

Mitochondria are the power plants of eukaryotic cells. They generate ATP for biochemical reactions and provide essential metabolites and signaling molecules for regulating cellular functions including cell death, lipid metabolism, and mitochondrial reactive oxygen species production [1, 2]. Cardiolipin (CL) is a dimeric phospholipid in which two phosphatidyl moieties are linked by a central glycerol backbone located at the mitochondrial inner membrane [3]. CL participates in apoptosis and respiratory complex stabilization [4] and is related to human diseases such as Barth syndrome, Alzheimer disease, and Parkinson disease [5–7].

Unlike other phospholipids, CL contains four acyl chains; this diversifies the possible number of CL species with regard to the combinations of fatty acyl chains. However, in the mammalian heart and skeletal tissues, the most common CLs are 18-carbon fatty acids, especially linoleic acid (LA), followed by the minor species stearic acid (18:0) and oleic acid (OA). The abundance of LA in CL in these tissues is maintained delicately [8], and its dysregulation is associated with aging and diseases. An animal study reported that compared with the heart of a young rat, the heart of an aged rat contained lower levels of LA-containing CL and higher levels of highly unsaturated fatty acids [9]. In addition, the tetralinoleoyl-CL level was found to be decreased in the heart tissue of rats with spontaneously hypertensive heart failure and in the left ventricle of patients with idiopathic dilated cardiomyopathy [10]. Tafazzin, a CL remodeling enzyme, was found to be mutated in patients with Barth syndrome whose tetralinoleoyl-CL levels were largely decreased and monolyso-CL levels were increased [7]. Nevertheless, LA supplementation increased CL levels and restored the decrease in tetralinoleoyl-CL levels in patients with Barth syndrome [11].

Dietary or lipid supplementation with different fatty acid species can modulate mitochondrial CL profiles by incorporating a fatty acyl moiety through remodeling and a biosynthesis pathway [12–14]. The human colorectal adenocarcinoma cell line HT-29 selectively incorporated exogenously supplemented docosahexaenoic acid (DHA, 22:6), LA (18:2), and OA (18:1) into CL [15]. The prostate cancer cell line PC-3 could incorporate supplemented fatty acids; CL levels were elevated by LA and diminished by arachidonic acid (AA) [15]. Treatment of neonatal cardiomyocytes and GL15 glioblastoma cells with palmitic acid caused the reduction of CL levels that affected the interaction of CL with cytochrome *c*, leading to apoptosis [16]. Regarding animal models, in rat heart myoblast H9c2 cells, supplemented polyunsaturated fatty acids, namely eicosapentaenoic acid (EPA, 20:5), DHA, and AA (20:4), could incorporate into CL [14]. In animal models, DHA potentially incorporated into CL by replacing the original LA in CL [17].

The chain length and saturation are two main characteristics of CL. The chain length of fatty acids affect the CL remodeling efficiency and the total carbon number of CL [18]. In our previous study, exogenous polyunsaturated fatty acids (PUFAs) changed the composition of fatty acyl chains and increased the unsaturation of CL [14]. The unsaturation level of lipids plays crucial roles in cellular physiology including membrane fluidity [19, 20], neurotransmitter release [21–24], and stemness maintenance in ovarian cancer stem cells [25, 26]. However, few studies have specifically analyzed the effects of fatty acid unsaturation on the CL structure and metabolism. Notably, 18-carbon fatty acyl chains are the most common fatty acid components in CLs. We hypothesized that supplementation of 18-carbon fatty acids with different numbers and positions of double bonds can result in their sequential incorporation into CL and affect the unsaturation of CL but not its chain length.

**Fig. 1:**
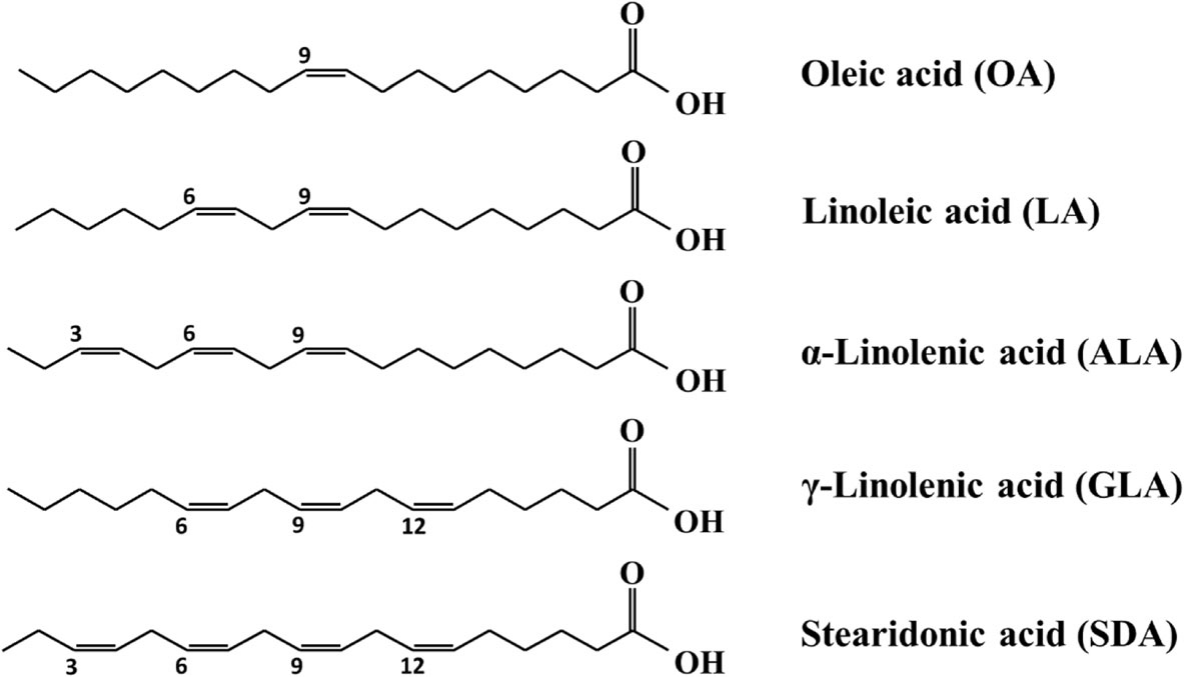
Structures of 18-carbon unsaturated fatty acids. The numbers above the fatty acids indicate the n-3 (ω-3), n-6 (ω-6), n-9 (ω-9), and n-12 positions of double bonds

Macrophage activation during inflammation triggered the production of mitochondrial reactive oxygen species (mtROS), leading to the formation of inflammasome [27]. Supplementation of the omega-3 and omega-6 fatty acids has been shown to trigger fatty acid incorporation into mitochondrial CL and regulate the inflammatory process [14, 28–30]. In this study, we supplied RAW264.7 cells with OA, LA, α-linolenic acid (ALA), γ-linolenic acid (GLA), and stearidonic acid (SDA). Subsequently, we measured CL species levels through liquid chromatography (LC)–mass spectrometry (MS) and evaluated the mRNA expression of lipid metabolism enzymes (Fig. 1). We noted that OA supplementation significantly increased the levels of CL72:3 and CL72:4; all supplemented fatty acids demonstrated a similar trend, but their effects on increasing both the number of double bonds in and the chain length of CL differed. Moreover, the supplemented fatty acids reduced the mRNA levels of *Fads2* and *Alox5* but increased that of *Cpt1a*.

## Materials and methods

### Materials

Dulbecco’s modified eagle medium (DMEM), fetal bovine serum (FBS), penicillin/streptomycin, 1 M HEPES and 0.5% trypsin-EDTA for cell culture were purchased from Gibco (Grand Island, NY, USA). Fatty-acid-free bovine serum albumin (BSA) was bought from Akron (Boca Raton, FL, USA). OA, LA, ALA, GLA and SDA were bought from Cayman (Ann Arbor, MI, USA). Primers were synthesized by ZGene Biotech Inc. (Taipei, Taiwan). iScript™ cDNA Synthesis kit and iQ™ SYBR® Green Supermix were acquired from Bio-Rad (Hercules, CA, USA). Invitrogen TRIzol reagent was purchased from Thermo Fisher Scientific (Waltham, MA, USA).

### Cell culture and fatty acid supplementation

RAW264.7 mouse macrophage cells were cultured in Dulbecco’s modified eagle medium with 10% fetal bovine serum, 0.5% penicillin/streptomycin and 25 mM HEPES in 5% CO_2_ at 37 °C. The unsaturated fatty acid OA, LA, ALA, GLA and SDA were selected for RAW264.7 supplementation. These fatty acids contain 18 carbons, which is the most abundant acyl chain length for RAW264.7, but have different number and positions of double bonds. The cells were seeded in 6-cm culture dish and supplemented with 200 μM OA, LA, ALA, GLA and SDA for 48 h. Unsaturated fatty acids were dissolved in BSA in 25 mM HEPES, and the fatty acid: BSA ratio was 6:1. All the experiments were performed in triplicates.

### Lipid extraction

The total lipids was extracted by using the Bligh-Dyer’s method [31]. After cells were collected, the 125 ng CL standard CL(14:0)_4_ was added to the cell pallets, and the samples were sonicated in ice bath in 2 mL methanol. Then, 1 mL of chloroform was added to the samples and vortexed for 10 min, which was done for two times. 1 mL of DDW was added into samples and further vortexed for 10 min. After centrifuging at 3000 rpm for 5 min, we collected the lower phase of the samples in the test tube and dried the samples using nitrogen gas.

### Mass spectrometry analysis

The extracted total lipids were re-dissolved in 200 μL of Acetonitrile/Isopropanol/H_2_O (63:30:5). 20 μL of the samples were analyzed by LC/MS Ion-trap (Bruker). There are two different HPLC mobile phases, including solution A: Acetonitrile:H_2_O (60:40), 10 mM ammonium formate, 0.1% formic acid and solution B: Isopropanol:Acetonitrile (90:10), 10 mM ammonium formate, 0.1% formic acid. Gradient was from 60% solution A to 100% solution B in 25 min and preserved in 100% solution B until 40 min and then backed to 60% solution A in an Acclaim RSLC 120 C18 2.1 mm × 100 mm × 2.2 μm column (Thermo, USA) at a flow rate of 0.2 mL/min at 55 °C. Then, we calculated the integrated area of the extract ion current (XIC) in Bruker DataAnalysis (ver.4.1). All experiments were in triplicate and the standard deviations of the triplicated experiments were plotted as the error bars of the figures.

### Total RNA extraction and reverse transcription

The cultured dishes were placed on ice for 5 min, then RAW264.7 cells were washed three times with ice-cold 1 mL PBS. After that, 1 mL TRIzol reagent was added to the cultured dishes for 5 min to isolate total RNA, and harvested the cell into eppendorf tube. The cell pallet was added 200 μL of chloroform, mixed evenly, and then centrifuged at 12000 rpm for 15 min at 4 °C. The top layer of the solution in the eppendorf tube was collected and added equal amount of isopropanol. The samples were kept at − 20 °C for 20 min, taken into room temperature for 10 min and then centrifuged at 12000 rpm for 15 min at 4 °C again. We washed the pellets with 500 μL ethanol and centrifuged at 9000 rpm for 5 min at 4 °C. The ethanol was eliminated thoroughly, and waited about 7 min to make sure that the pellets were totally dried. After adding 20 μL DEPC water (RNase free), we heated the samples to 50 °C for 5 min. The mRNA was stored at − 80 °C. Using iScript™ cDNA Synthesis Kit to proceed in reverse transcription, the 1 μg total RNA was mixed with 4 μL of 5x iScript reaction mix and 1 μL of iScript reverse transcriptase, then made up to 20 μL of total volume with additional nuclease-free water. The mixture was placed at 25 °C for 5 min, heated at 46 °C for 20 min, inactivated the reverse transcriptase at 95 °C for 1 min and stored at − 20 °C eventually.

### RT-qPCR

The 50 ng cDNA template was mixed with 10 μL of iQ™ SYBR® Green Supermix and 500 nM of forward and reverse primers (Table 1) in qPCR tube, then added DNase-free H_2_O to 20 μL of total volume. Before starting the thermal cycling program, we centrifuged the samples to ensure that the air bubbles were removed. The reaction was first initiated at 95 °C for 3 min to activate polymerase and denature the DNA on MiniOpticon Real-Time PCR System (Bio-Rad, Hercules, CA, USA). Then, the cDNA was amplified at 95 °C for 15 s and the combinational annealing-extension was at 60 °C for 90 s for 40 cycles. All experiments are in triplicate and the error bars are standard deviation of the triplicated experiments.

**Table 1:** Primers of the RT-qPCR

Gene	Accession Number	Forward primer 5′-3′	Reverse primer 5′-3′
Gapdh	NM_008084	AACTTTGGCATTGTGGAAGG	GGATGCAGGGATGATGTTCT
Cds1	NM_173370	GGAGAGACGGTGGCAGATTA	CAGGTAGAGGGCGAAGGATA
Pgs1	NM_133757	ACGCTGATTGGCTCTCCTAA	CTGCTCTTGCTCCTGATGAA
Lclat1	NM_001081071	ACCGCCTAAGAGAAGGGAAG	TGGATGTGGGAAGAGAGTCA
Taz	NM_​001173547	CCTTATCACCGTCTCCAACC	GTCCAACGCATCAACTTCAG
Pla2g6	NM_001199023	GAGACTGCCTTCCATTACGC	TCAGCCCTTGGTTGTTTACC
Pnpla8	NM_26164	TTCCTTTCTCGTCCCACTGA	GCAGACACTTCCTGTTCTTCG
Fads2	NM_019699	CAAAACCAACCACCTGTTCTT	AAAGGCTGTGACGAGGGTAG
Pld6	NM_001290283	CTCTGCCTCTTCGCCTTCT	ACCTGTATCCCTGCCTTGC
Cycs	NM_007808	AAATCTCCACGGTCTGTTCG	TGCCCTTTCTCCCTTCTTCT
Bid	NM_007544	AGCCCTTGATGAGGTGAAGA	GCAAAGATGGTGCGTGACT
Plscr3	NM_001168497	CTGGACTTGTGGCTGTGGTA	GGCATCTGTGAGGGCTTCT
Ptgs1	NM_008969	ACAGTGCGGTCCAACCTTAT	AGAGGGCAGAATGCGAGTAT
Ptgs2	NM_011198	CCCCCACAGTCAAAGACACT	ATCATCAGACCAGGCACCA
Alox5	NM_009662	CATCAAGAGCAGGGAGAAGC	CATAGTTGGAGGAGCGTTGG
Fads2	NM_019699	CAAAACCAACCACCTGTTCTT	AAAGGCTGTGACGAGGGTAG
Cpt1a	NM_013495	ATGGACTCTAGTGATACA	ATCTCTGGATGTAGTAGG
Cpt2	NM_009949	CCAGTTCAGGAAGACAGA	CGAGCAGTTAAATACATATCAAA

### Statistical analysis

Volcano plot was created by plotting the fold changes of each CL and MLCL species in fatty acid supplementation compare to control group against the *p*-value calculated from the comparison between fatty acid supplementation and control group. Both fold changes and p-value were shown in log scale for clear presentation. The statistical analyses were performed by two-sided unpaired Student’s t-test and calculated by Microsoft Excel. The statistical significance of differences was set at *p* < 0.05. The error bars of the results are the standard deviation of three independent experiments.

## Results

### CL profile shifted on MS spectrum

Eighteen-carbon fatty acids are the most common fatty acids in mammalian cells. We hypothesized that fatty acid unsaturation affects the efficiency of CL biosynthesis and CL remodeling-related enzymes and then changes the function of mitochondria. Thus, 18-carbon fatty acids with different numbers and positions of double bonds were added to RAW264.7 cells and then cultured for 48 h. We collected the cells and extracted the total lipid for MS analysis. Because stearic acid could not be thoroughly dissolved in the aqueous solution of BSA, we did not include this fatty acid in our study to avoid an inconsistent concentration and results. Among the supplemented unsaturated fatty acids, OA is a ω-9 fatty acid, LA and GLA are ω-6 fatty acids, and ALA and SDA are ω-3 fatty acids.

**Fig. 2:**
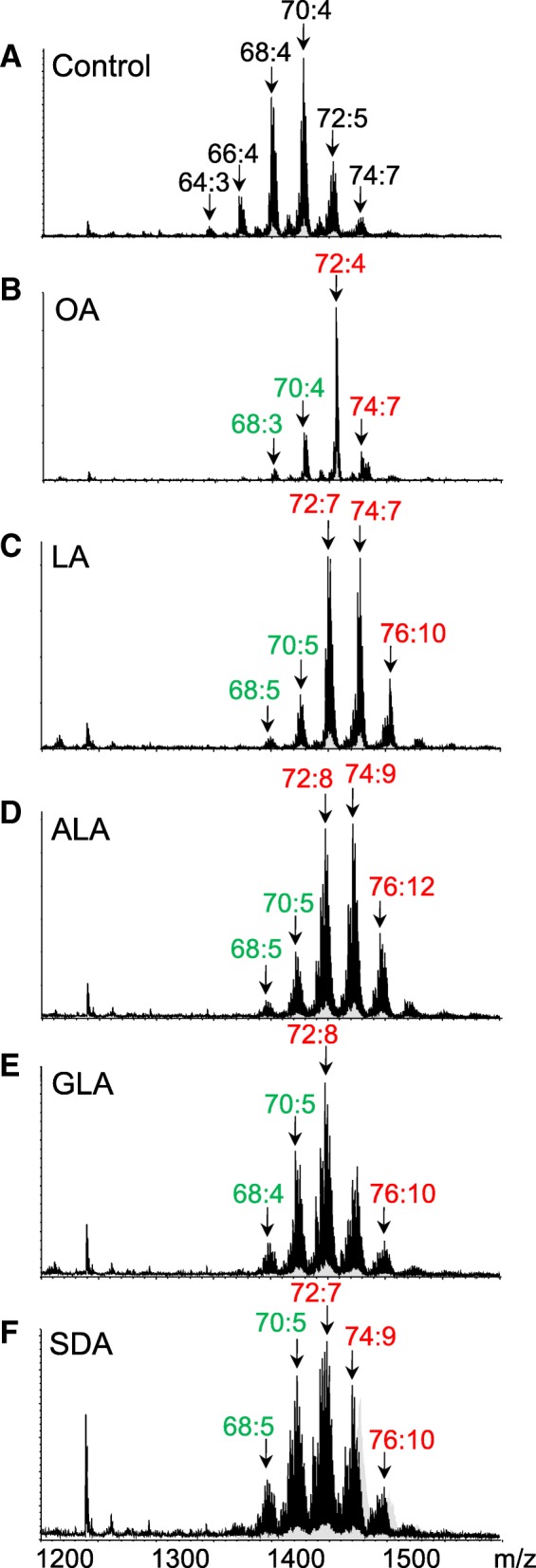
Cardiolipin (CL) species in RAW 264.7 cell supplemented with different fatty acids. Mass spectra of CL obtained from RAW 264.7 cell treated with (**a**) BSA (control), and (**b**) oleic acid (OA), **c** linoleic acid (LA), **d** α-linolenic acid (ALA), **e** γ-linolenic acid (GLA), and (**f**) stearidonic acid (SDA). All fatty acids are conjugated with BSA for supplementation. Major CL species in each mass cluster were indicated as total carbon number and double bonds of fatty acyl moieties in each CL species. Red and green represents the percentage increased and decreased compare to control group

In RAW264.7 cells, CL displayed as groups on the mass spectrum (Fig. 2a). The five main groups of CL were distributed between *m/z* 1300 and 1500, representing 66, 68, 70, 72, and 74 carbons in fatty acyl moieties. In each CL group, different numbers of double bonds were noted at a 2-Da difference. For clear expression, we used either CL(72:4) and MLCL(54:3) to demonstrate the total number of carbons and double bonds in fatty acyl moieties or CL(18:1)_4_ and MLCL(18:1)_3_ to indicate specific acyl chains.

Supplementation of the five 18-C fatty acids differentially changed the CL spectrum. OA(18:1) supplementation considerably changed the distribution pattern of the mass envelope to a pattern with the major CL72 group (Fig. 2b). CL72:4 (CL(18:1)_4_) in the CL72 group became the most abundant species. When LA(18:2) was supplemented, the overall CL mass envelope shifted to a higher mass range (Fig. 2c). We estimated a four-carbon addition of CL from the changes of the mass spectrum. Further supplementation of ALA (18:3) resulted in a similar mass shift pattern as LA(18:2) (Fig. 2d). The high mass CL76 and CL78 species showed a similar increase as did the LA(18:2) treatment. Because CL76 and CL78 contain 20-C fatty acyl chain, this result indicates that both ALA and LA can be elongated after entering the cell. We also noticed that the width of the mass envelope significantly increased, indicating the increase in the levels of CL species in each CL group. Although GLA and ALA are 18:3 fatty acids, they have one different double bond at either 6 or 15 position. With the difference of the double bond positions, GLA supplementation resulted in a different pattern than ALA supplementation did (Fig. 2e). The mass also shifted to a higher mass range after ALA treatment, but their increasing level was less significant than that after GLA supplementation. Finally, the mass envelope demonstrated a significant shift after SDA(18:4) supplementation. However, the distribution of mass peaks was similar to that after GLA supplementation, even though SDA contains one more double bond than GLA does (Fig. 2f). Overall, the width of the mass group peak widened when high double bond fatty acids were supplemented.

### Quantification of CL after fatty acid supplementation

**Fig. 3:**
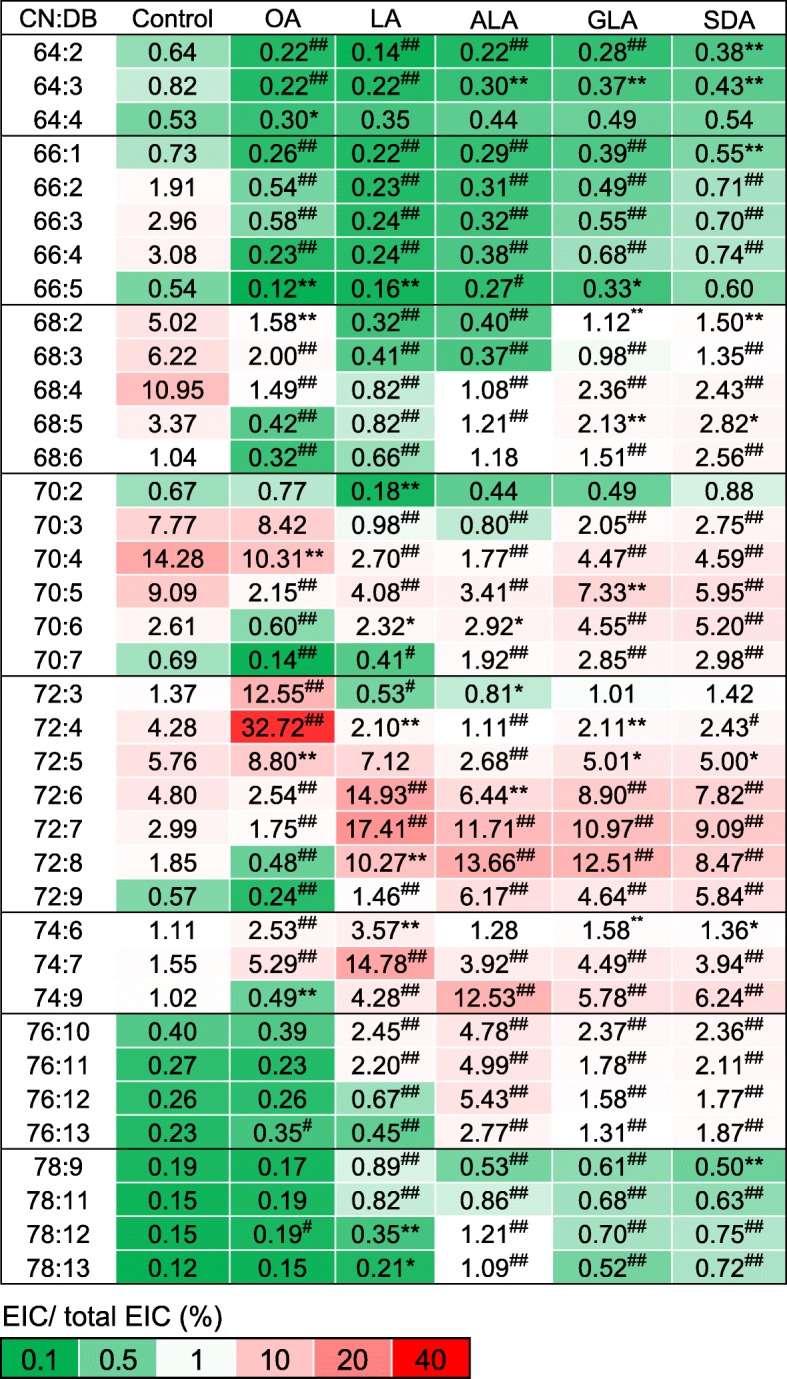
Heat map of the percentage of individual CL of total CL pool in RAW 264.7cell treated with BSA (control), OA, LA, ALA, GLA, and SDA. CN:DB indicates total carbon number and double bonds of fatty acyl moieties in each cardiolipin species. All fatty acids are conjugated with BSA for supplementation. Mean, *p < 0.05, ^#^*p* < 0.01, **p < 0.005, ^##^*p* < 0.001 vs. Control. *N* = 3

To relatively quantify each CL species, we added a CL standard, CL(14:0)_4_, into each sample as a reference, which is *m/z* 1239.7. Based on the extract ion current, the relative quantitation herein was shown as percentages (Fig. 3). The major CL contained 70 carbons in fatty acyl moieties. CL(70:4) was the most abundant species in untreated RAW264.7 cells. We previously determined the fatty acyl compositions of CL in RAW264.7 cells through MS/MS [32], demonstrating that most fatty acyl moieties contain 16, 18, and 20 carbons with 0, 1, and 2 double bonds.

OA supplementation condensed CL to CL72 group. CL72:4, CL72:3, and CL72:5 were the most abundant species shown in the heat map, accounting for a total of 54.0%. The most significant changes in CL species, CL72:3 and CL72:4, were composed of CL(18:0) (18:1)_3_ and CL(18:1)_4_, respectively, thus indicating the incorporation of OA. For LA supplementation, the calculated percentage changes revealed that CL68 and CL70 shifted to CL72 and CL74, respectively. The number of double bonds in CL72 and CL74 increased from 4 to 7, implying the incorporation of two 18:2 into CL to replace the original 16:0 or 16:1. Notably, we also observed the increase in the levels of CL76 and CL78, which are composed of a mixture of 18- and 20-carbon acyl chains. Because 20-carbon fatty acids were not supplied to the cells, our results strongly suggest that 18:2 fatty acids were further elongated to longer fatty acyl chains for CL remodeling. The condensation effects on CL72 sequentially dissipated when the cells were supplemented with ALA, GLA and SDA, leading to the increased width of the mass envelope. The level of double bond-abundant species, such as CL72:7, CL72:8, CL72:9, and CL74:9, showed a significant percentage increase after ALA, GLA and SDA supplementation.

When we compared the total CL content in CL76 after GLA and ALA supplementation, the increasing percentage decreased by more than 50% in GLA, indicating that GLA is a less favored fatty acid for elongation. However, SDA supplementation did not significantly increase the number of double bonds in CL. The desaturation effects of GLA and SDA were similar. The groups demonstrating major changes were CL74 and CL76. Based on the results of GLA and SDA, the double bond at C6 position may limit the elongation of fatty acids for CL remodeling.

**Fig. 4:**
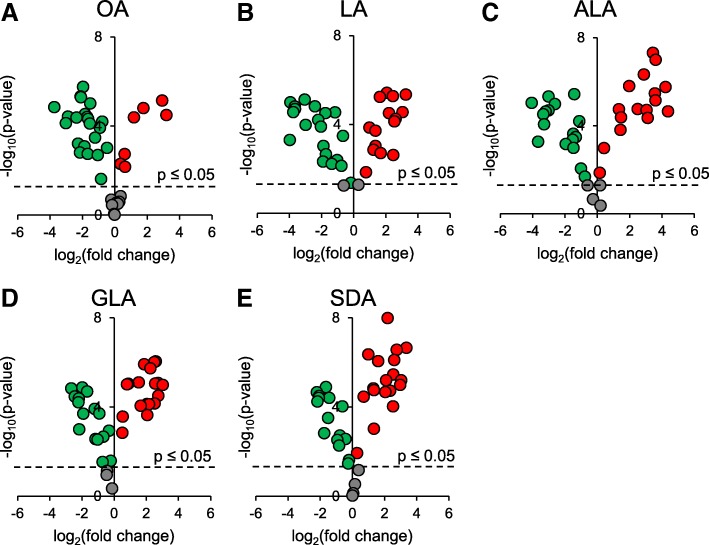
Volcano plot of the changes of CL species in RAW 264.7cell supplemented with (**a**) OA (**b**) LA (**c**) ALA (**d**)GLA and (**e**) SDA compare to BSA-treated RAW. All fatty acids are conjugated with BSA for supplementation. Red and green dots represent the CL species that are significantly increased and decreased compare to control group, respectively

For comprehensive analysis, the significance and the fold changes were analyzed by volcano plot (Fig. 4). The *p*-values of the fold-change increase CL of the five treatments positively depended on the number of double bonds of the supplemented fatty acids. The reverse trends could be observed in the fold-change decrease CL. In other words, high number of double bond in the supplemented fatty acids highly deviated CL remodeling. Not only the intensity of the significance, the number of the significant increasing CL also positively depended on the number of double bond. The reverse trends of the decreasing CL were again observed. It is particularly worth to note that ALA supplementation showed high fold-change the other fatty acid supplementation.

### Fatty acids triggered changes in MLCL species

**Fig. 5:**
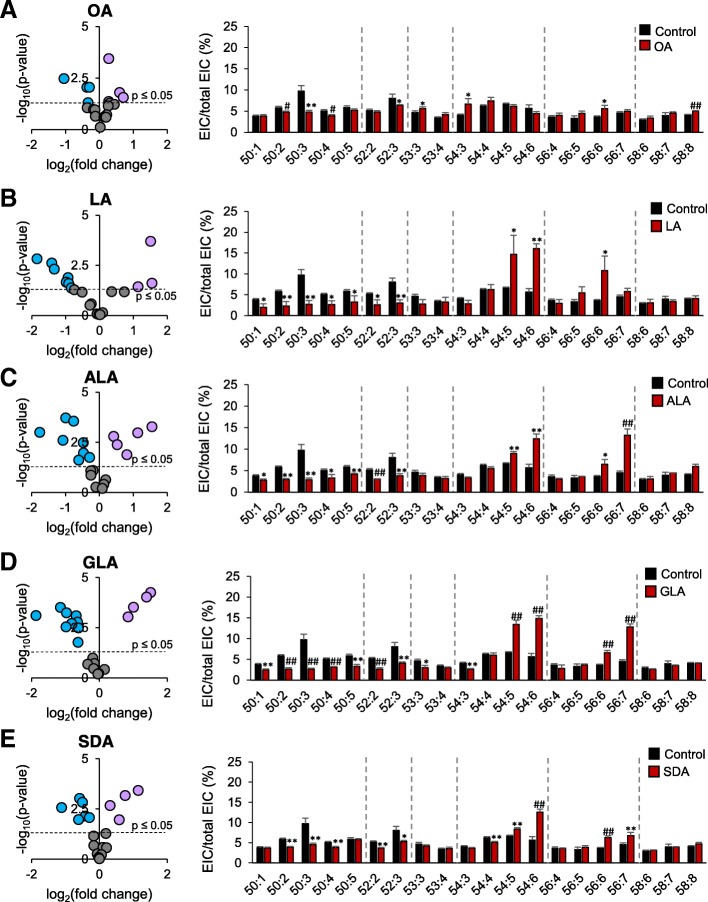
MLCL species in RAW 264.7 cell supplemented with fatty acids. Volcano plot of the changes of MLCL species and comparison of MLCL molecular in RAW 264.7cells supplemented with (**a**) OA (**b**) LA (**c**) ALA (**d**) GLA, and (**e**) SDA. All fatty acids are conjugated with BSA for supplementation. Mean ± SD, *p < 0.05, #*p* < 0.01, **p < 0.005, ##*p* < 0.001 vs. control group (BSA). *N* = 3

The MLCL most consistently changed by 18-carbon fatty acids was in group C54 (Fig. 5). In this group, C54 is composed of three fatty acyl chains with C18 carbon. Supplementation of the higher double bond-containing fatty acids also increased the changes in C56. The volcano test showed that 18:2 and 18:3 had a relatively sparse distribution, followed by 18:4. The 18:1 supplementation did not substantially change the MLCL pattern as the other 18-carbon fatty acids did.

### Fatty acids affected elongation and transportation genes

**Fig. 6:**
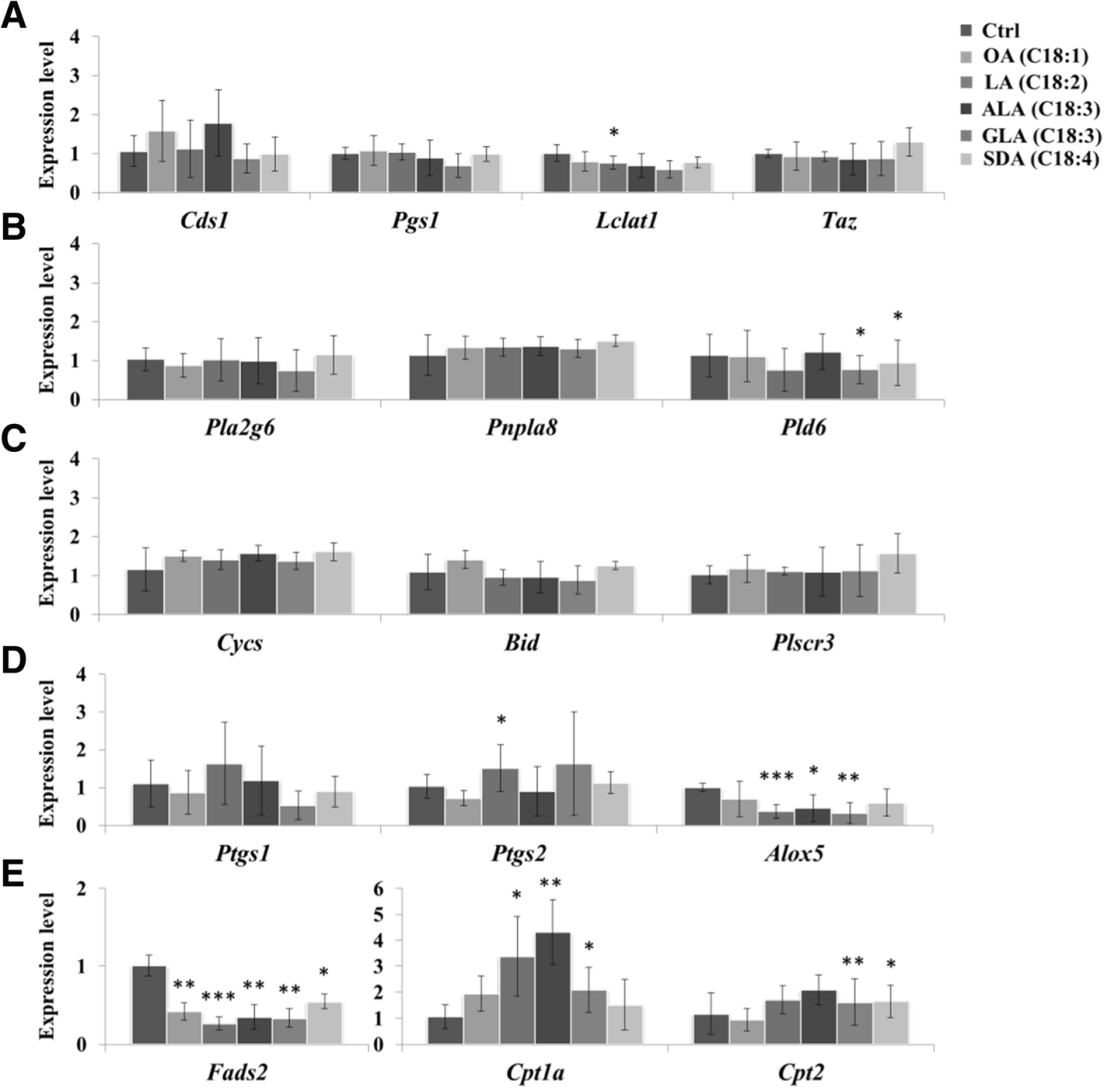
Gene expression affected by supplemented unsaturated fatty acids. The gene expression of the OA-, LA-, ALA-, GLA-, or SDA-treated RAW264.7 cells were quantified by real-time PCR. The selected genes were all involved in CL metabolism, including (**a**) CL synthesis and remodeling genes (**b**) CL degradation genes (**c**) apoptosis genes (**d**) inflammation genes, and (**e**) fatty acid elongation and transportation genes. The expression levels were normalized to the control cells. The error bars are the standard deviations of the triplicated experiments

The MS results revealed that the unsaturation level of fatty acids could differentially change the compositions of CL, which further affected the membrane structure of mitochondria. To understand whether and how fatty acid supplementation triggers CL remodeling through gene expression, we further analyzed the gene expression of CL metabolism-related genes through real-time PCR after fatty acid supplementation. Here, we focused on genes related to CL biosynthesis (*Cds1* and *Pgs1*), CL remodeling (*Taz* and *Lclat1*), CL degradation (*Pla2g6*, *Pnpla8* and *Pld6*), CL externalization (*Plscr3*), apoptosis (*Cycs* and *Bid*), inflammation (*Ptgs1*, *Ptgs2* and *Alox5*), fatty acid desaturation (*Fads2*) and fatty acid transportation (*Cpt1a* and *Cpt2*) (Fig. 6). Notably, fatty acid supplementation did not affect the expression of CL biosynthesis, remodeling, and degradation genes; it also did not trigger apoptosis. We found that the supplementation of 18-carbon fatty acids reduced *Alox5* expression, indicating that the exogenous fatty acid inhibited *Alox5* expression (Fig. 6d). LA and GLA could be converted to AA, which might cause feedback inhibition. The trend of *Fads2* was similar to that of *Alox5* upon fatty acid supplementation (Fig. 6e), indicating that the supplementation of these fatty acids might limit the function of desaturases through gene regulation. Without the proper function of desaturase, the fatty acid elongation would be limited. The import of excess fatty acids would be accelerated into mitochondria through carnitine-acyl transferase I, which is a highly expressed enzyme (Fig. 6e).

## Discussion

The incorporation of the supplementary fatty acids to CL involves multiple cellular processes. The fatty acids can incorporate into cellular membranes as fatty acyl chains of phospholipids [33, 34] and further into mitochondrial membranes [14, 32]. For an incorporated fatty acid molecule, an exogenous fatty acid may encounter elongation, desaturation, mitochondrial import process, beta-oxidation, phospholipid synthesis, and CL remodeling. Besides the analysis of the changes of CL by mass spectrometry, we investigated how the CL incorporation process was regulated through gene transcription by RT-qPCR. We first evaluated gene expression of the fatty acid desaturation and elongation by *Fads2*, and the import to mitochondria by *Cpt1a* and *Cpt2*. After transported into mitochondria, the fatty acid may stimulate the synthesis of phosphatidylglycerol(PG) by *Pgs1* and Cytidine Diphosphate Diacylglycerol CDP-DAG by *Cds1* for nascent CL synthesis, or the CL remodeling through *Taz* and Lclat1 to produce mature CL. Excess or damaged CL can be degraded though the regulation of gene expression of *Pla2g6*, *Pnpla8* and *Pld6*. *Cycs*, *Bid* and *Plscr3* were critical for mitochondria function, apoptosis and CL externalization. The inflammatory gene markers, *Ptgs1*, *Ptgs2* and *Alox5* were also evaluated.

**Fig. 7:**
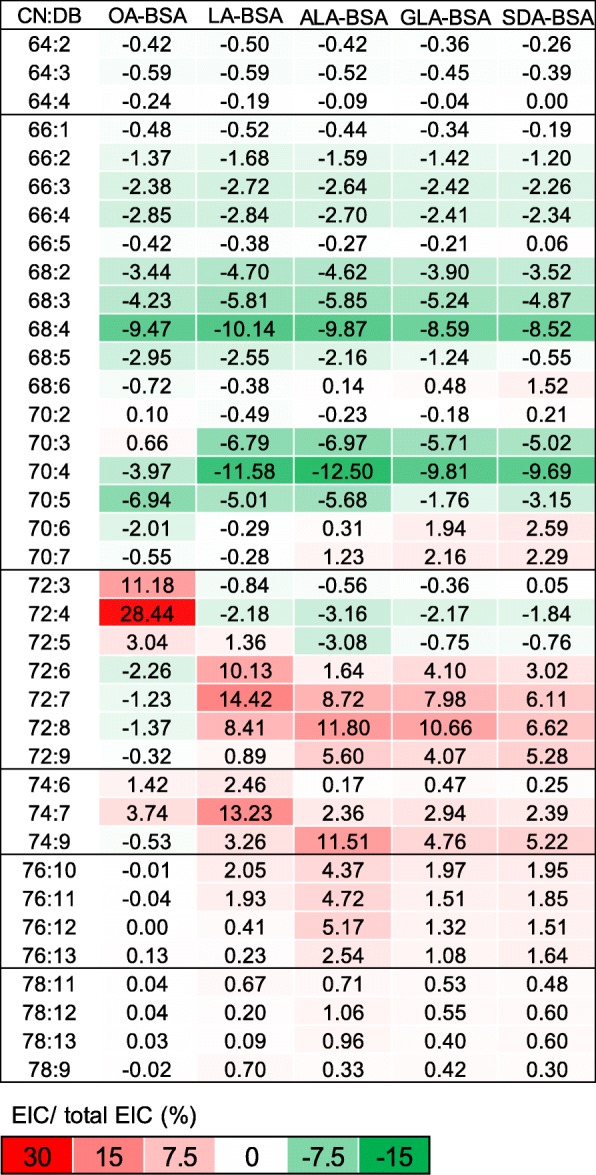
Differential heat map of the changes of cardiolipin species supplemented with OA, LA, ALA, GLA, and SDA. The fold changes represented the ratio between fatty acid treatment and the control, which was shown on the heat map as the color index. Red color means that the result is at least more than 7.5%-fold, and the green color indicates that the result is at least less than − 7.5%-fold

In this study, the effects of fatty acid supplementation on CL species were evaluated using a heat map (Fig. 7). There is a high probability that these 18-carbon fatty acids were used to remodel CL without further modification. The 16-carbon acyl chains of the short-chain CL groups CL64, CL66, CL68, and CL70 were replaced by 18-carbon fatty acyl moieties; thus, the percentages of these groups decreased. By contrast, the percentage of the long-chain remodeled CL groups CL72, CL74, CL76 and CL78 increased. The CL72 group, which contains four 18-carbon moieties, evidently showed that the unsaturation of an exogenous fatty acid corresponded to an increase in the number of double bonds in CL. Some species in CL72 showed more than 10% increases.

Because CL72 is mainly composed of 18-carbon fatty acyl chains [32], the supplementation of external 18-carbon fatty acids significantly altered the CL72 species. Direct incorporation of fatty acids could be observed after supplementation of OA (18:1). Among the CL species demonstrating a large increase, the content of CL72:4, containing four 18:1 acyl chains, increased by 28%. According to our data, CL72:3, CL70:4, and CL70:3, containing three 18:1 fatty acyl chains, showed minor, but significant, increases. Considering specificity, OA (18:1) supplementation tended to affect CL72:3 and CL72:4, indicating an efficient incorporation process of OA (18:1) to mitochondrial CL.

Synthesis of PUFAs, such as AA, EPA, and DHA, requires further elongation and desaturation of these 18-carbon fatty acids [35], including OA, LA, ALA [36–38], SDA on the ER and/or mitochondria. Here, *Fads2* expression was downregulated to inhibit the desaturation of long-chain fatty acid after supplementation of these 18 carbon fatty acids. LA (18:2) and ALA (18:3) are good substrates for fatty acid desaturase 2 (FADS2). We indeed observed that *Fads2* was most downregulated by LA (18:2), followed by ALA (18:3). This downregulation indicates that the supplementation of 18-carbon fatty acid surpassed the needs of PUFAs in cells and therefore caused a shutdown of fatty acid elongation and desaturation. Inhibition of the delta-6 desaturase has been shown to reverse CL remodeling and prevents contractile dysfunction in the heart of the aged mouse [39]. Therefore, the desaturases are critical for the regulation of fatty acid incorporation to CL.

Direct incorporation of LA and ALA into CL could also be observed. LA supplementation resulted in 10, 14, and 8% increases in CL72:6, CL72:7, and CL72:8, respectively, and 13% on CL74:7, all of which contain two or more 18:2 fatty acyl chains, indicating direct incorporation of LA (18:2). After LA and ALA supplementation, the most remodeled or replaced CL species were CL70:4 and CL68:4, with one average double bond in one of the four acyl chains showing approximately 10% decreases on average. LA (18:2) and ALA (18:3) tended to increase both the number of double bonds and the chain length of various CL species, unlike OA (18:1), which specifically targeted CL72:4. On comparing the effects of LA (18:2) and ALA (18:3) on the CL72 group, we noted that the direct incorporation of fatty acids was eased in ALA, indicating that LA (18:2) is a favored substrate for CL remodeling; however, excessive LA (18:2) inhibited fatty acid elongation. Although the inhibition of *Fads2* expression was noted, fatty acid elongation was minor. Less inhibition of *Fads2* expression by ALA (18:3) also resulted in an increase in the levels of CL76, which may contain more than two fatty acyl moieties that have been elongated (Fig. 7). In particular, the high number of double bonds in CL76:10, CL76:11, CL76:12, and CL76:13 species was coordinately detected after ALA (18:3) treatment.

Because 18:4 did not show the most downregulation of *Fads2* expression, this downregulation is not dependent on the total number of double bonds; however, it slightly favors 18:2. *Cpt1a* expression was upregulated to accelerate the import of more 18:2 and 18:3, but fewer 18:1 and 18:4, PUFAs into mitochondria, indicating that the purpose of this upregulation was not beta-oxidation. For beta-oxidation, the higher energetic 18:1 would have been the best energy source. The two species, 18:2 and 18:3, are potentially required for other synthesis and therefore stimulate the transport through mitochondrial membrane.

GLA (18:3) and SDA (18:4) both contain the n-15 double bond, which is the least favored for CL remodeling. GLA and SDA generated the least fatty acid incorporation profile in all five supplemented fatty acids. GLA and SDA both had fewer effects on *Fads2* downregulation and *Cpt1a* upregulation, particularly SDA. The effects of SDA supplementation on the CL profile are also limited. Because of the high number of double bonds, SDA (18:4) and GLA (18:3) addition also highly diversifies CL species, as observed on the mass spectrum.

OA and LA have higher specificity for CL incorporation, which can change the mitochondrial activities. Supplementation of PG(18:1)_4_ and PG(18:2)_4_ on RAW264.7 had shown similar mitochondrial CL incorporation effects [32]. Without macrophage activation, the mitochondrial activities were not affected. Once the macrophage was KDO2-Lipid A (KLA)-activated, the fatty acid-modified mitochondria can resist from the KLA-induced mitochondrial activity loss. Furthermore, 18 carbons are the most common CL acyl chains in various species. This is the same in RAW264.7 cells, but not as dominant as the heart and liver tissues, where most CLs are in symmetrical CL forms [32]. Symmetrical CL containing either 18:1 or 18:2 is critical for maturation [40]. However, the species in other tissues and most cell lines, including RAW cells, are shown in groups. The reasons and causes of the CL symmetry require further clarification. The saturation of fatty acids affects the hydrophobicity and structure of fatty acids, as well as CL, which may cause the specificity of these fatty acids in CL remodeling. In the experiments, we observed the saturation effects of exogenous 18-carbon fatty acids on CL remodeling. Here, 18:1 was a good substrate of the remodeling enzyme tafazzin, which transfers supplementary fatty acids to generate symmetrical CL72:4. The substitution likely targeted the short-chain acyl chain, and the number of double bonds was not the priority. Therefore, the 16-carbon fatty acyl chain became the first target for 18:1 and 18:2 for the exchange. From the pattern, CL70 and CL72 were converted from CL68 and CL70, respectively. Few species of CL72 containing 20-carbon fatty acyl chains may convert to CL74. Although the levels of symmetrical CL(18:2)_4_ increased, there was only approximately 10% increase, which is still a low percentage of symmetrical CL. We also noted that the overall increase in CL74 levels after 18:2 supplementation was much higher than that after 18:1 supplementation. Therefore, it is highly possible that some originally supplemented 18:2 were elongated to 20-carbon species. When ALA (18:3) was supplemented, this elongation effect become prominent, but the effect was not as significant as that after GLA supplementation.

Fatty acid supplementation affects fatty acid elongation, desaturation, and transportation and triggers changes in CL species, but none of these CL changes are through the gene regulation of CL remodeling- or synthesis-related genes. We did not observe any regulation of downstream CL degradation-related genes. Furthermore, 18-carbon fatty acid supplementation neither triggered apoptosis nor elevated eicosanoid production. Our data suggest that 18:1 is the most efficient fatty acid to incorporate into CL to form symmetrical CL without elongation and desaturation. Moreover, 18:2 and 18:3 can be further elongated before incorporation. These fatty acids significantly increase the number of double bonds and the chain length of CL. GLA and SDA may be unsuitable remodeling enzyme substrates.

## Conclusions

Fatty acid supplementation activated desaturation of fatty acid and its transportation into mitochondria in macrophage. The double bonds of fatty acids changed the CL incorporation pathway. OA(18:1) was incorporated directly; LA(18:2) and ALA(18:3) were desaturated and elongated before remodeling; GLA(18:3) and SDA(18:4) were poorly incorporated into mitochondrial CL.

## Abbreviations

AA: Arachidonic acid
ALA: α-linolenic acid
CL: Cardiolipin
DHA: Docosahexaenoic acid
EPA: Eicosapentaenoic acid
GLA: γ-linolenic acid
LA: Linoleic acid
OA: Oleic acid
PUFAs: Polyunsaturated fatty acids
SDA: Stearidonic acid
